# Choline-Sigma-1R as an Additional Mechanism for Potentiation of Orexin by Cocaine

**DOI:** 10.3390/ijms22105160

**Published:** 2021-05-13

**Authors:** Jeffrey L. Barr, Pingwei Zhao, G. Cristina Brailoiu, Eugen Brailoiu

**Affiliations:** 1 Center for Substance Abuse Research, Lewis Katz School of Medicine at Temple University, Philadelphia, PA 19140, USA; Jeffrey.Barr@SanfordHealth.org (J.L.B.); zhaopw@temple.edu (P.Z.); 2 Department of Pharmaceutical Sciences, Jefferson College of Pharmacy, Thomas Jefferson University, Philadelphia, PA 19107, USA; Gabriela.Brailoiu@jefferson.edu

**Keywords:** choline, orexin A, OX_1_ receptor, phospholipase D, PLD, reward

## Abstract

Orexin A, an endogenous peptide involved in several functions including reward, acts via activation of orexin receptors OX_1_ and OX_2_, Gq-coupled GPCRs. We examined the effect of a selective OX_1_ agonist, OXA (17-33) on cytosolic calcium concentration, [Ca^2+^]_i_, in neurons of nucleus accumbens, an important area in the reward circuit. OXA (17-33) increased [Ca^2+^]_i_ in a dose-dependent manner; the effect was prevented by SB-334867, a selective OX_1_ receptors antagonist. In Ca^2+^-free saline, the OXA (17-33)-induced increase in [Ca^2+^]_i_ was not affected by pretreatment with bafilomycin A1, an endo-lysosomal calcium disrupter, but was blocked by 2-APB and xestospongin C, antagonists of inositol-1,4,5-trisphosphate (IP_3_) receptors. Pretreatment with VU0155056, PLD inhibitor, or BD-1047 and NE-100, Sigma-1R antagonists, reduced the [Ca^2+^]_i_ response elicited by OXA (17-33). Cocaine potentiated the increase in [Ca^2+^]_i_ by OXA (17-33); the potentiation was abolished by Sigma-1R antagonists. Our results support an additional signaling mechanism for orexin A-OX_1_ via choline-Sigma-1R and a critical role for Sigma-1R in the cocaine–orexin A interaction in nucleus accumbens neurons.

## 1. Introduction

Orexin A and B (also known as hypocretin-1 and -2) are endogenous neuropeptides synthesized in hypothalamic neurons that control appetite, sleep/wakefulness, hormone release, stress, and drug-seeking behavior [1,2,3]. Hypothalamic neurons expressing orexins project to several brain areas such as ventral tegmental area, nucleus accumbens, dorsal raphe nucleus, and locus coeruleus [2,4].

Orexins act via OX_1_ and OX_2_ receptors, Gq-coupled GPCRs that may signal also via Gs or Gi proteins [5,6]. OX_1_ receptors have a preferential role in addiction, reward, and motivation, while OX_2_ receptors are involved in arousal [3,7]. OX_1_ receptor activation leads to an increase in cytosolic Ca^2+^ concentration, [Ca^2+^]_i_, subsequent to activation of phospholipase C (PLC) and generation of inositol-1,4,5-trisphosphate (IP_3_) [6,8]. In addition to the PLC coupling, activation of OX_1_ receptor leads to phospholipase D (PLD) activation [9,10]. PLD-mediated hydrolysis of phosphatidylcholine produces choline and phosphatidic acid [11]. Choline activates Sigma-1R [12], a chaperone protein residing at the endoplasmic reticulum that potentiates IP_3_-induced Ca^2+^ release [13].

OX_1_ receptors were identified in brain nuclei from the reward circuit, including nucleus accumbens [14,15], and OX_1_-selective antagonists have been evaluated as potential therapeutic agents for addiction treatment [16,17,18]. Previous studies indicate that orexins via OX_1_ receptor activation are involved in the response to cocaine and play multiple roles in cocaine addiction-related behaviors [7,19,20,21]. Orexin-OX_1_ signaling is required for stimulant locomotor sensitization and cocaine seeking when it is driven by highly motivated states [3]. OX_1_ receptors in the nucleus accumbens mediate chronic cocaine-induced locomotor sensitization [22]. Other studies indicate that SB-334867, a selective OX_1_ receptor antagonist, prevents cocaine seeking and is a potential treatment target for cocaine relapse prevention [23].

Cocaine acts primarily by blocking the dopamine transporter, thus increasing dopamine transmission in the nucleus accumbens [24], an important area in the reward circuit [25]. In addition, cocaine binds to and activates Sigma-1R [26]. Since nucleus accumbens neurons express Sigma-1R [27,28,29] and OX_1_ receptors [14,15] and behavioral studies support the cocaine–orexin interaction at this level [3,22], in this work, we examined the underlying mechanisms and role of Sigma-1R in the cocaine–orexin interaction in nucleus accumbens neurons.

## 2. Results

### 2.1. OXA (17-33) Increases Cytosolic Ca^2+^, [Ca^2+^]_i_, in Nucleus Accumbens Neurons via OX_1_ Receptor Activation

**Figure 1:**
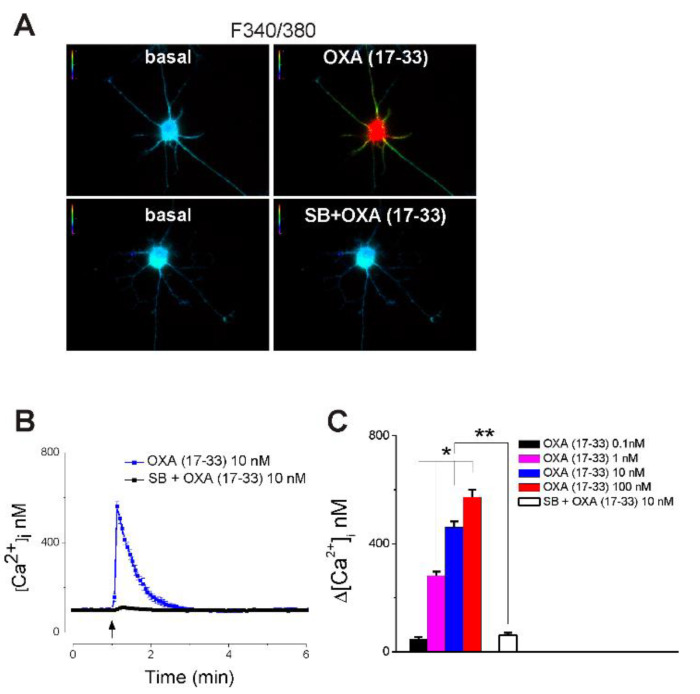
OXA (17-33) increases cytosolic Ca^2+^ concentration, [Ca^2+^]_i_, in nucleus accumbens neurons via OX_1_ receptor activation. (**A**) Representative examples of fluorescence F340/380 ratio of Fura-2AM-loaded nucleus accumbens neurons in basal conditions (left) and after treatment with OXA (17-33) (10 nM), a selective OX_1_ agonist, alone (top right) or in the presence of OX_1_ antagonist, SB-334867 (1 µM) (bottom right). (**B**) OXA (17-33) (10 nM) produced a transient increase in [Ca^2+^]_i_; the effect was abolished by SB-334867. (**C**) Comparison of the amplitude of [Ca^2+^]_i_ increase (mean + SD) produced by OXA (17–33) (0.1, 1, 10, and 100 nM); *p* < 0.05 as compared with the amplitude of [Ca^2+^]_i_ increase produced by each concentration (*) or by OXA (17-33) (10 nM) (**); *n* = 6 neurons/each concentration tested.

OXA (17-33) (0.1–100 nM), i.e., truncated orexin A, a selective OX1 agonist [1,30], increased [Ca^2+^]_i_ in nucleus accumbens neurons in a dose-dependent manner (Figure 1). OXA (17-33) (10 nM) increased the fluorescence F340/380 ratio of Fura-2AM-loaded nucleus accumbens neurons; the effect was prevented by pretreatment with SB-334867 (1 µM), a selective OX_1_ antagonist [31] (Figure 1A). OXA (17-33) (10 nM) produced a transient increase in [Ca^2+^]_i_ that was abolished by SB-334867 (Figure 1B). Comparison of the amplitude of the increase in [Ca^2+^]_i_ produced by different concentrations of OXA (17-33) (0.1, 1, 10, 100 nM) is illustrated in Figure 1C (*n* = 6 neurons/each concentration). Of note, 20–30 neurons were tested for each condition, and an increase in [Ca^2+^]_i_ was identified in about 25% of neurons tested; the amplitude of [Ca^2+^]_i_ from the response of six neurons was used for analysis.

### 2.2. OXA (17-33) Increases [Ca^2+^]_i_ via IP_3_-Dependent Mechanism

**Figure 2:**
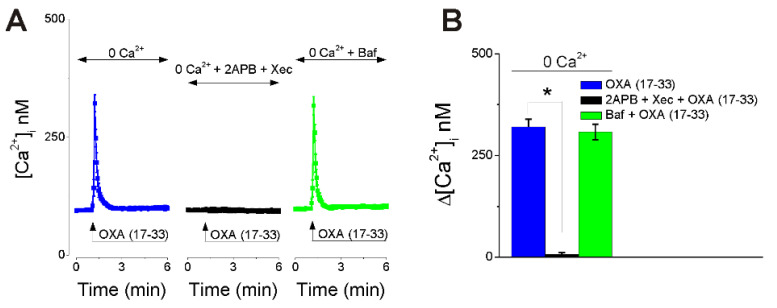
OXA (17-33) increases [Ca^2+^]_i_ via IP_3_-dependent mechanism. (**A**) Illustration of average Ca^2+^ transients (± SD) induced in Ca^2+^-free saline by OXA (17-33) (10 nM) alone (left) and OXA (17-33) (10 nM) after pretreatment with 2-aminoethoxydiphenyl borate (2-APB, 100 µM) and xestospongin C (XeC, 10 µM, 15 min), IP_3_ receptor antagonists (middle), or with bafilomycin A1 (Baf, 1 µM) (right). (**B**) Comparison of the amplitude of the increase in [Ca^2+^]_i_ (average + SD) in each condition. Pretreatment with 2-APB and xestospongin C abolished the Ca^2+^ response induced by OXA (17-33). * *p* < 0.05; *n* = 6 neurons/condition.

In Ca^2+^-free saline, OXA (17-33) (10 nM) elicited an increase in [Ca^2+^]_i_ of lower amplitude (Figure 2) than in Ca^2+^-containing saline (Figure 1). The Ca^2+^ response to OXA (17-33) (10 nM) in Ca^2+^-free saline was abolished by pretreatment with IP_3_ receptors antagonists 2-aminoethoxydiphenyl borate (2-APB, 100 µM, 15 min) and xestospongin C (10 µM, 15 min) [32], indicating a PLC-dependent mechanism. Disruption of lysosomal Ca^2+^ stores with bafilomycin A1 (1 µM, 1 h preincubation), a V-type ATPase inhibitor that prevents lysosomal acidification [33], did not affect the Ca^2+^ response to orexin (10 nM) (Figure 2). OXA (17-33) (10 nM)-induced Ca^2+^ responses (average ± SD) in Ca^2+^-free saline in nucleus accumbens neurons in the absence and presence of 2-APB and xestospongin C or bafilomycin A1 are illustrated in Figure 2A, and a comparison of the amplitude of the [Ca^2+^]_i_ increase in each condition is illustrated in Figure 2B (*n* = 6 neurons/condition).

### 2.3. OXA (17-33) Increases [Ca^2+^]_i_ via Choline-Sigma-1R-Dependent Mechanism

**Figure 3:**
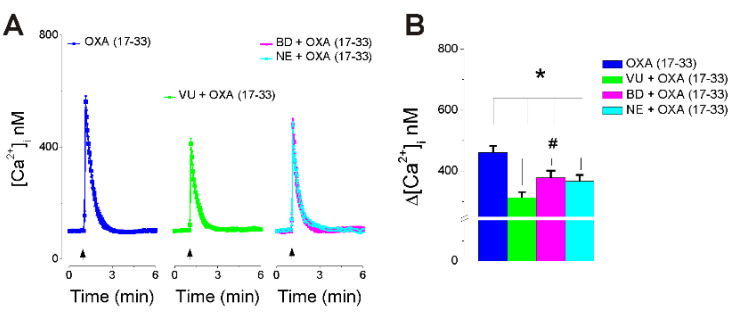
OXA (17-33) A increases [Ca^2+^]_i_ via choline-Sigma-1R-dependent mechanism. (**A**) Illustration of average Ca^2+^ transients (± SD) induced by OXA (17-33) (10 nM) alone (left) and in the presence of VU0155056 (1 μM), PLD inhibitor (middle), and BD1047 (50 µM) or NE-100 (5 µM), Sigma-1R antagonists (right). (**B**) Comparison of the amplitude of the increase in [Ca^2+^]_i_ (average + SD) in each condition. Inhibition of PLD or antagonism of Sig-1R reduces the Ca^2+^ response elicited by OXA (17-33) (10 nM); *p* < 0.05 as compared to amplitude of [Ca^2+^]_i_ increase produced by OXA (17-33) (*) or produced in the presence of the inhibitors (^#^) (*n* = 6 neurons/condition).

Pretreatment with VU0155056 (1 μM, 30 min), a PLD inhibitor [34], reduced the amplitude of OXA (17-33) (10 nM)-induced increase in [Ca^2+^]_i_ by 33% (Figure 3). Pretreatment with BD1047 (50 µM, 30 min) or NE-100 (5 µM, 30 min) (Sigma-1R antagonists) [35,36] reduced the Ca^2+^ response to OXA (17-33) (10 nM) by 18.1% and 20.4%, respectively. Average Ca^2+^ responses induced by OXA (17-33) alone and in the presence of PLD inhibitor and Sigma-1R antagonists are illustrated in Figure 3A, and a comparison of the amplitude of the [Ca^2+^]_i_ increase in each condition is illustrated in Figure 3B (*n* = 6 neurons/condition).

### 2.4. Cocaine Potentiates OXA (17-33)-Induced Increase in [Ca^2+^]_i_ via Sigma-1R Activation

**Figure 4:**
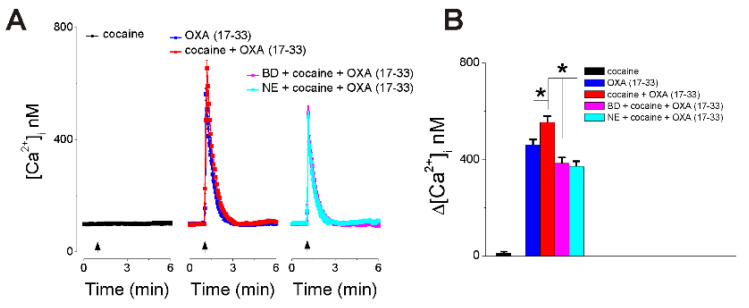
Cocaine potentiates OXA (17-33)-induced increase in [Ca^2+^]_i_ via Sigma-1R activation. (**A**) Illustration of average Ca^2+^ responses (± SD) produced by application of cocaine (10 µM) alone (left, no response), cocaine (10 µM) and OXA (17-33) (10 nM) (middle), and cocaine and OXA (17-33) in the presence of Sigma-1R antagonists BD1047 (50 µM) or NE-100 (5 µM) (right). (**B**) Comparison of the amplitude of the increase in [Ca^2+^]_i_ (average + SD) in each condition. Cocaine potentiates the Ca^2+^ response induced by OXA (17-33), while antagonism of Sigma-1R abolished the potentiation produced by cocaine on the Ca^2+^ response elicited by OXA (17-33) (10 nM). * *p* < 0.05 (*n* = 6 neurons/condition).

Cocaine (10 µM), while it did not elicit a Ca^2+^ response by itself, potentiated the increase in [Ca^2+^]_i_ produced by OXA (17-33) (10 nM), when added at the same time as OXA (17-33) (Figure 4). Pretreatment with BD1047 (50 µM, 30 min) or NE-100 (5 µM, 30 min), Sigma-1R antagonists, reduced the increase in [Ca^2+^]_i_ produced by cocaine + OXA (17-33) (10 nM), by 30.7% and 33.1%, respectively (Figure 4). This indicates that antagonism of Sigma-1R abolished the potentiation produced by cocaine and further reduced the Ca^2+^ response to OXA (17-33) (10 nM) to the same level as in neurons treated with Sigma-1R antagonists before OXA (17-33) alone (Figure 4 vs. Figure 3). A comparison of the amplitude of the [Ca^2+^]_i_ increase in each condition is illustrated in Figure 4B (*n* = 6 neurons/condition).

**Figure 5:**
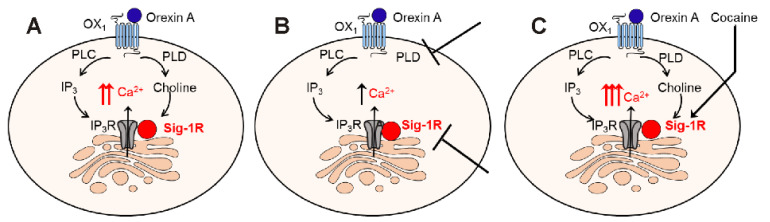
Diagram illustrating the proposed model of potentiation of orexin by cocaine via Sigma-1R in nucleus accumbens neurons. (**A**) Orexin A acting on OX_1_ receptor activates PLC and PLD. PLC increases IP_3_ level and promotes the Ca^2+^ release from endoplasmic reticulum via IP_3_ receptors (IP_3_R). PLD produces choline (from hydrolysis of phosphatidylcholine) that acts on Sigma-1R to potentiate Ca^2+^ increase via IP_3_ R (higher increase in [Ca^2+^]_i_). (**B**) Inhibition of PLD or antagonism of Sigma-1R limits the orexin A-OX_1_ receptor signaling to PLC-mediated IP_3_-dependent increase in Ca^2+^ (smaller increase in [Ca^2+^]_i_). (**C**) Cocaine, via Sigma-1R activation, potentiates the PLC- and PLD-mediated increase in [Ca^2+^]_i_ produced by orexin A acting on OX_1_ (highest increase in [Ca^2+^]_i_). The diagram was created using the Motifolio Illustration Toolkit Neuroscience (https://www.motifolio.com accessed on 7 March 2021).

A diagram summarizing the proposed mechanism of potentiation of orexin by cocaine via Sigma-1R activation in nucleus accumbens neurons is illustrated in Figure 5.

## 3. Discussion

Orexin A, via activation of OX_1_ receptor, can activate both phospholipase C (PLC) and phospholipase D (PLD) in various cell models [9,10] including neurons [37]. PLC activation leads to hydrolysis of phosphoinositides and formation of inositol-1,4,5- trisphosphate (IP3), the Ca^2+^-releasing second messenger that releases Ca^2+^ from endoplasmic reticulum (ER) through IP_3_ receptors [38]. PLD activation promotes the hydrolysis of phosphatidylcholine to choline and phosphatidic acid [11]. Whereas phosphatidic acid was considered the main effector downstream to PLD activation, we recently identified choline as a second messenger that activates Sigma-1R [12].

Sigma-1 receptor is a chaperone protein expressed in the endoplasmic reticulum (ER), mainly at the mitochondria-associated ER membrane domains (MAMs) [13]. Sigma-1Rs interact with many different signaling proteins. At the ER, Sigma-1Rs potentiate the Ca^2+^ release via IP_3_ receptors [13]; they also interact with STIM1, the Ca^2+^ sensor for store-operated Ca^2+^ entry [39]. Sigma-1R ligands include antidepressants, antipsychotics, and drugs of abuse [40]. Cocaine, in addition to its canonical target that elevates synaptic dopamine levels, binds to and activates Sigma-1Rs [41,42]. Neurons in the nucleus accumbens, a key area involved in the reward circuit [25], express Sigma-1R [27,28,29] and OX_1_ receptors [14,15]. Behavioral studies supported the cocaine–orexin interaction in nucleus accumbens [3,22], but the underlying mechanisms remained unclear; this prompted us to investigate the mechanisms of cocaine–orexin interaction at this level.

Orexin A has been reported to increase cytosolic Ca^2+^ concentration, [Ca^2+^]_i_, in various cells expressing orexin receptors [1], including neurons [43]. We first tested the effect of truncated orexin A peptide, OXA (17-33), a selective OX1 agonist [30], on [Ca^2+^]_i_ in cultured nucleus accumbens neurons. OXA (17-33) increased [Ca^2+^]_i_ in a dose-dependent manner; the effect was abolished by SB-334867 (1 µM), an OX_1_ antagonist [10,31] indicating that it was mediated by OX_1_ receptors.

We next demonstrated that the OXA (17-33)-induced increase in [Ca^2+^]_i_ was mediated by IP_3_-dependent Ca^2+^ release from ER, as previously reported [6]; the effect was abolished by IP_3_ receptor antagonists, but not affected by disruption of lysosomal Ca^2+^ stores.

In other series of experiments, pretreatment with PLD inhibitor reduced the Ca^2+^ response elicited by OXA (17-33), supporting the involvement of PLD activation in addition to PLC/IP_3_-dependent mechanisms in nucleus ambiguus neurons. This is in agreement with previous studies reporting PLD-dependent mechanisms downstream to OX1 activation [9,10,37].

In addition, antagonism of Sigma-1R reduced the Ca^2+^ response produced by OXA (17-33), indicating for the first time the role of Sigma-1R in the response to OX_1_ activation in the nucleus accumbens. The reduction in the response to OXA (17-33) produced by PLD inhibition and Sigma-1R antagonism indicates that choline produced by PLD hydrolysis of phosphatidylcholine, acting on Sigma-1R, as recently reported [12], potentiates the IP_3_-mediated increase in [Ca^2+^]_i_.

Our results also indicate that cocaine, while it did not elicit a response by itself, potentiated the increase in [Ca^2+^]_i_ induced by OXA(17-33). This is similar to the potentiation of orexin A-induced increase in [Ca^2+^]_i_ by cocaine reported in VTA neurons [43]. In VTA neurons, the effect of orexin and the potentiation by cocaine were abolished by suvorexant (MK-4305), a dual orexin receptor OX_1_/OX_2_ antagonist [43,44]. Moreover, in nucleus accumbens neurons, the potentiation of orexin response by cocaine was abolished by Sigma-1R antagonists. Cocaine is a Sigma-1R agonist [26], and we previously reported that, in nucleus accumbens neurons, cocaine via Sigma-1R potentiates the IP_3_-mediated increase in [Ca^2+^]_i_ [29]. Here, we identify an additional signaling mechanism for orexin A–OX_1_ via choline-Sigma-1R and a critical role for Sigma-1R in the cocaine–orexin A interaction in nucleus accumbens neurons.

## 4. Materials and Methods

### 4.1. Chemicals

OXA (17-33), i.e., truncated orexin A, a selective OX1 agonist [30], SB-334867, a selective nonpeptide OX_1_ antagonist [31], and BD-1047 and NE-100 (Sigma-1 antagonists) were obtained from Tocris (Bio-Techne Corporation, Minneapolis, MN, USA). VU0155056, a PLD inhibitor [34], was purchased from Avanti Polar Lipids (Alabaster, AL, USA). Cocaine was supplied by the National Institute on Drug Abuse’s Drug Supply Program. All other chemicals were from Sigma Aldrich (St. Louis, MO, USA), unless otherwise mentioned.

### 4.2. Neuronal Cell Culture

Nucleus accumbens neurons were dissociated from neonatal Sprague Dawley rats (Ace Animal Inc., Boyertown, PA, USA) of both sexes as previously described [29,45]. Newborn rats were decapitated, and the brains quickly removed surgically and immersed in ice-cold Hanks balanced salt solution (HBSS). The nucleus accumbens was identified, removed, minced, and subjected to enzymatic (papain, 37 °C) and mechanical dissociation. Cells were cultured in Neurobasal A medium (Life Technologies, ThermoFisher Scientific, Carlsbad, CA, USA) containing 10% fetal bovine serum, 1% GlutaMax, and 1% penicillin–streptomycin–amphotericin B solution at 37 °C in a humidified atmosphere with 5% CO_2_.The mitotic inhibitor cytosine β-arabinofuranoside (1 µM) was added to the culture to inhibit glial cell proliferation. For calcium imaging, neurons were cultured on round 25 mm diameter glass coverslips coated with poly-l-lysine, in six-well plates.

### 4.3. Measurement of Cytosolic Ca^2+^ Concentration

Cytosolic Ca^2+^ concentration, [Ca^2+^]_i_, was measured by calcium imaging methods in nucleus accumbens neurons loaded with Fura-2AM, as previously described [29,45]. Cells were incubated with 5 µM Fura-2AM (Invitrogen) in HBSS at room temperature for 45 min, in the dark, and then incubated for another 45 min in HBSS to allow for complete de-esterification of the dye. Coverslips (25 mm diameter) were subsequently mounted in an open bath chamber (Warner Instruments, Hamden, CT, USA) on the stage of an inverted microscope Nikon Eclipse TiE (Nikon Inc., Melville, NY, USA), equipped with a Perfect Focus System and a Photometrics CoolSnap HQ2 CCD camera (Photometrics, Tucson, AZ, USA). During the experiments, the Perfect Focus System was activated. Fura-2AM fluorescence (emission = 510 nm), following alternate excitation at 340 and 380 nm, was acquired at a frequency of 0.25 Hz. Images were acquired and analyzed using NIS-Elements AR software (Nikon Inc.). After appropriate calibration with ionomycin and CaCl_2_ and with Ca^2+^ free and EGTA, respectively, the ratio of the fluorescence signals (340/380 nm) was converted to Ca^2+^ concentrations [46].

### 4.4. Data Analysis

Data were expressed as the mean ± standard deviation (SD). Datasets were compared for statistically significant differences using one-way ANOVA followed by post hoc Bonferroni test. A *p*-value <0.05 was considered statistically significant.

## Data Availability

The data generated and analyzed during this study are available in the manuscript.
